# Rivaroxaban versus enoxaparin for the prevention of recurrent venous thromboembolism in patients with cancer: A meta-analysis

**DOI:** 10.1097/MD.0000000000011384

**Published:** 2018-08-03

**Authors:** Jiali Xing, Xiangbao Yin, Desheng Chen

**Affiliations:** a Queen Mary Institute, School of Medicine, Nanchang University; b Department of Hepatobiliary and Pancreatic Surgery, the Second Affiliated Hospital of Nanchang University, Nanchang; c Emergency Department, the People's Hospital of Ningdu County, Ningdu, Jiangxi, China.

**Keywords:** bleeding, cancer, enoxaparin, rivaroxaban, thrombosis

## Abstract

**Background:**

Although low-molecular-weight heparin (LMWH) is recommended as the first-line treatment in patients with active cancer and venous thromboembolism (VTE), many patients are more willing to choose oral anticoagulants. We collected currently available data to evaluate the efficacy and safety of the oral direct factor Xa inhibitor rivaroxaban compared with enoxaparin in patients with cancer and VTE.

**Methods:**

We retrieved electric databases, including Medline/PubMed and EMBASE, from inception through January, 2018. We included articles comparing enoxaparin with rivaroxaban in patients with cancer and VTE. Recurrences of VTE, incidence of major bleeding and deaths were compared between groups. Poole analysis was conducted in Review Manager Version 5.2.

**Results:**

A total of 4 articles and 667 patients were included in the final analysis. Pooled analysis showed that rivaroxaban was associated with a non-significantly lower recurrence of VTE (risk ratio [RR] = 0.55, 95% confidence interval (95%CI): 0.28–1.06, *I*^2^ = 0%). Patients treated with rivaroxaban had a similar major bleeding risk compared with those administrated with enoxaparin (RR = 0.84, 95%CI: 0.39–1.83, *I*^2^ = 0%). No significant difference was observed in mortality between the 2 groups (RR = 0.51, 95%CI: 0.15–1.80, *I*^2^ = 89%).

**Conclusion:**

Rivaroxaban is as effective and safe as enoxaparin for the prevention of recurrent VTE in patients with malignancy. Rivaroxaban is a potential option for patients with cancer and VTE.

## Abstract 2

Supplemental Digital Content is available in the text

## 1 Introduction

Venous thromboembolism (VTE) is a very common comorbidity in patients with cancer. It is known that VTE comprises pulmonary embolism (PE) and deep venous thrombosis (DVT), and approximately 10% to 20% patients with PE or VTE have either active cancer or a history of cancer.^[1,2]^ In patients with active cancer and VTE, low-molecular-weight heparin (LMWH) is recommended as the first-line treatment.^[3]^ However, LMWH injections are inconvenient and patients are more willing to choose oral anticoagulants. Warfarin, the most used oral vitamin K antagonists (VKAs), has its natural drawbacks, such as frequent serum monitoring of international normalized ratio, high risk of bleeding,^[4]^ and less time in therapeutic range.^[5]^ Thus, new anticoagulation strategies for patients with cancer and VTE are needed. The advent of newer factor Xa inhibitors such as rivaroxaban might bring a new option.

Rivaroxaban has been shown to be as effective as VKAs for the treatment and prevention of VTE in the general population.^[6]^ The number of cancer patients involved in previous studies is relatively small,^[7–9]^ and evidence comparing rivaroxaban with LMWH remains limited. In this study, we collected currently available data to evaluate the efficacy and safety of rivaroxaban compared with enoxaparin, a most used LMWH, in patients with cancer and VTE.

## 2 Methods

An ethical approval is not necessary as this is a meta-analysis. We performed this study in accordance with the PRISMA statement.^[10]^

### 2.1 Data sources and selection

We retrieved electric databases, including Medline/PubMed and EMBASE, from inception through January, 2018. The following terms were used in the search process: cancer or carcinoma and rivaroxaban. We also scanned the references lists of searched papers for potential eligible articles. No language restriction was applied.

According to the predefined criteria, we included articles comparing enoxaparin with rivaroxaban in patients with cancer and VTE. Conference abstracts were excluded.

### 2.2 Data extraction

Two independent researchers extracted baseline data, including the number of patients, ages, and body mass index. Outcomes of interest in each group, such as recurrences of VTE (including DVT and PE), major bleeding, and deaths were recorded. Disagreements were resolved by consulting a third party.

Quality assessment of included studies was conducted using the Newcastle-Ottawa Scale.

### 2.3 Statistics

Risk ratios (RRs) and 95% confidence intervals (95%CIs) were calculated in Review Manager Software (Version 5.2; The Cochrane Collaboration, Oxford, United Kingdom), using the method of Mantel–Haenszel. The value of *I*^2^ was used to show the heterogeneity across studies, a random-effects model was applied if *I*^2^ >50%. Funnel plot was used to indicate the publication bias. *P* < .05 was taken as statistically significant.

## 3 Results

### 3.1 Baseline characteristics

According to our predefined criteria, a total of 4 articles and 667 patients were included in the final analysis^[8,9,11,12]^ (Flow Diagram). The majority of included articles (3 out of 4) were published in 2017. There were 369 and 298 patients in rivaroxaban and enoxaparin group, respectively. As shown in Table 1, the mean age of included patients was around 60 years, and median treatment duration ranged from 110 to 204 days.

**Table 1:**
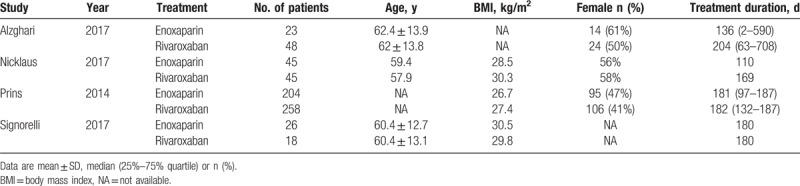
Baseline characteristics of included studies and subjects.

### 3.2 Recurrence of VTE

**Figure 1:**
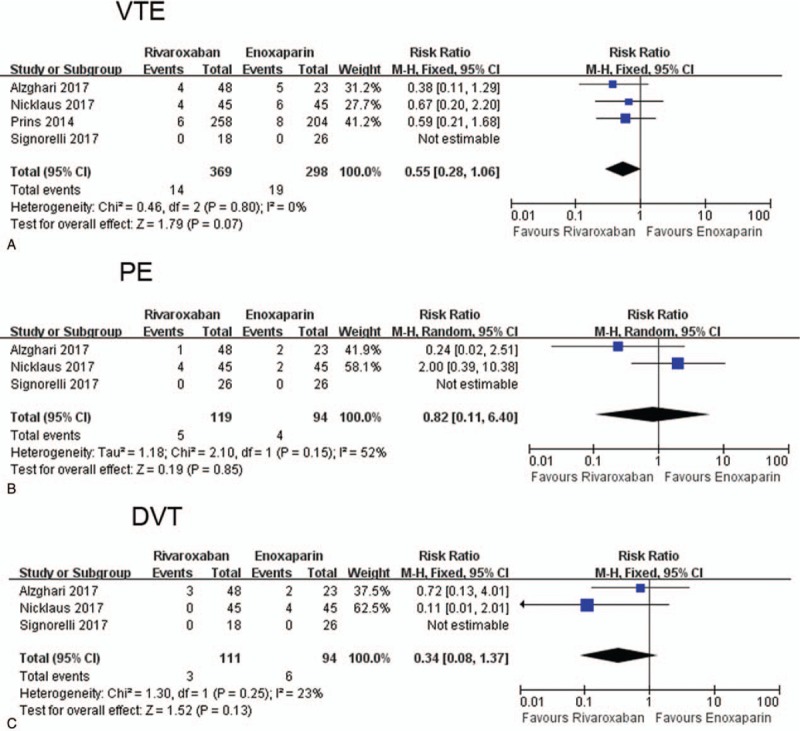
Forest plot comparing the recurrence of VTE (A), PE (B), and DVT (C) in rivaroxaban and enoxaparin group. DVT = deep venous thrombosi, PE = pulmonary embolism, VTE = venous thromboembolism.

The recurrence of VTE was 14 out of 369 (3.8%) and 19 out of 298 (6.4%) in rivaroxaban and enoxaparin group, respectively. Pooled analysis showed that rivaroxaban was associated a non-significantly lower recurrence of VTE (RR = 0.55, 95%CI: 0.28–1.06, *I*^2^ = 0%) (Fig. 1A). When dividing VTE to DVT and PE, we found that the recurrence of PE (RR = 0.82, 95%CI: 0.11–6.40, *I*^2^ = 52%) or DVT (RR = 0.34, 95%CI: 0.08–1.37, *I*^2^ = 23%) was also similar in rivaroxaban and enoxaparin group (Fig. 1B and C). Funnel plot did not show significant publication bias (Supplementary Figure 1A).

### 3.3 Major bleeding

**Figure 2:**
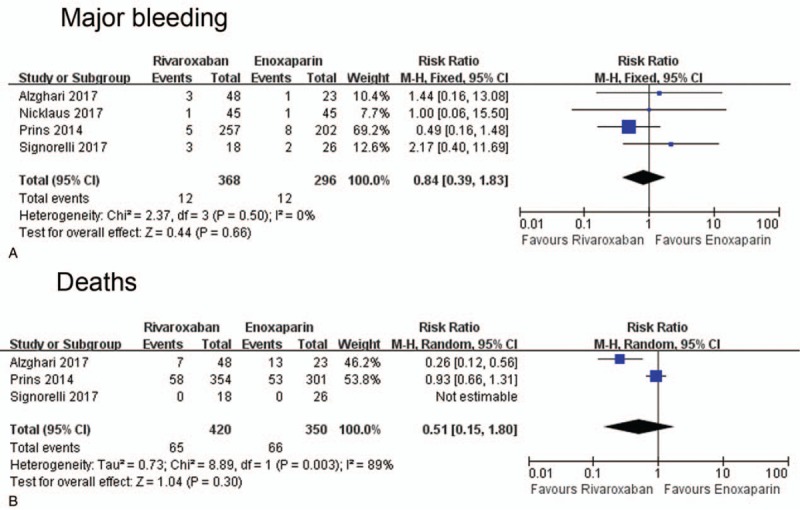
Forest plot comparing the incidence of major bleeding (A) and mortality (B) in rivaroxaban and enoxaparin group.

The incidence of major bleeding was 3.3% (12 out of 368) and 4.1% (12 out of 296) in rivaroxaban and enoxaparin group, respectively. Pooled analysis indicated that patients treated with rivaroxaban had a similar major bleeding risk compared with those administrated with enoxaparin (RR = 0.84, 95%CI: 0.39–1.83, *I*^2^ = 0%) (Fig. 2A). Funnel plot did not show significant publication bias (Supplementary Figure 1B).

### 3.4 Mortality

The mortality was 15.5% and 18.9% in rivaroxaban and enoxaparin group, respectively. No significant difference was observed in mortality between the 2 groups (RR = 0.51, 95%CI: 0.15–1.80, *I*^2^ = 89%) (Fig. 2B). No significant publication bias was detected in funnel plot (Supplementary Figure 1C).

## 4 Discussion

This study comprises a relatively large number of selected patients and demonstrates that rivaroxaban is as effective and safe as enoxaparin for the prevention of recurrent VTE in patients with cancer.

VTE is an independent risk factor for mortality and an important cause of death in patients with malignancy.^[13]^ The disadvantages of LMWH and warfarin make new oral anticoagulants such as rivaroxaban an attractive option for cancer patients with VTE. However, there is a lack of strong evidence in favor of rivaroxaban administration in patients with cancer. To our best knowledge, this is the first meta-analysis comparing rivaroxaban to LMWH in patients with malignancy.

Our analysis showed that there was a trend towards decreased recurrent VTE in cancer patients treated with rivaroxaban compared those with enoxaparin. Although the difference did not reach statistical significance due to the limitation of sample size, we found that all the endpoints, including recurrence of VTE, incidence of major bleeding, and mortality were non-significantly lower in rivaroxaban group than in enoxaparin group. It is plausible to believe that future data will provide stronger evidence. However, in this stage, we may conclude that rivaroxaban is at least not inferior to LWMH for treatment and prevention of VTE in cancer patients, which offers an additional choice for patients cannot adhere to injections.

Our paper comprises several limitations. First, it was a meta-analysis based on observational studies. In the absence of a randomized controlled trial in this topic, our work might represent the highest level of evidence to date, and call for future large, randomized, well-designed trials to provide new insight. Second, the sample size was relatively small, with only 4 articles being included in the final analysis. So the result should be interpreted with some caution. Third, age is an important factor to predict risk of VTE. However, previous studies included have not compared the mean age of patients with VTE and active cancer with that of patients only with VTE. Future studies should take consider of this issue. Last, different types and stages of cancers will affect the results and conclusion of this study. However, not all included studies have classified the types or stages of cancers, and the limited sample size precluded us to conduct further sub-group analysis. More large, well-designed studies are needed to explore this issue.

## 5 Conclusion

Rivaroxaban is as effective and safe as enoxaparin for the prevention of recurrent VTE in patients with malignancy. Rivaroxaban is a potential option for patients with cancer and VTE.

## Author contributions

**Conceptualization:** Xiangbao Yin.

**Data curation:** Xiangbao Yin.

**Formal analysis:** Xiangbao Yin.

**Investigation:** Jiali Xing, Desheng Chen.

**Methodology:** Jiali Xing.

**Resources:** Xiangbao Yin.

**Software:** Desheng Chen.

**Supervision:** Xiangbao Yin.

**Writing – original draft:** Jiali Xing.

**Writing – review and editing:** Xiangbao Yin, Desheng Chen.

## Supplementary Material

Supplemental Digital Content

## References

[1] Silverstein MD Heit JA Mohr DN Trends in the incidence of deep vein thrombosis and pulmonary embolism: a 25-year population-based study. Arch Intern Med 1998;158:585–93. 9521222 10.1001/archinte.158.6.585

[2] Buller HR Davidson BL Decousus H Subcutaneous fondaparinux versus intravenous unfractionated heparin in the initial treatment of pulmonary embolism. N Engl J Med 2003;349:1695–702. 14585937 10.1056/NEJMoa035451

[3] Streiff MB Holmstrom B Ashrani A Cancer-associated venous thromboembolic disease, Version 1.2015. J Natl Compr Canc Netw 2015;13:1079–95. 26358792 10.6004/jnccn.2015.0133

[4] Prandoni P Lensing AW Piccioli A Recurrent venous thromboembolism and bleeding complications during anticoagulant treatment in patients with cancer and venous thrombosis. Blood 2002;100:3484–8. 12393647 10.1182/blood-2002-01-0108

[5] Lee AY Levine MN Baker RI Low-molecular-weight heparin versus a coumarin for the prevention of recurrent venous thromboembolism in patients with cancer. N Engl J Med 2003;349:146–53. 12853587 10.1056/NEJMoa025313

[6] Nunnelee JD Review of an article: oral rivaroxaban for symptomatic venous thromboembolism. The EINSTEIN Investigators et al. N Engl J Med 2010; 363(26):2499-2510. J Vasc Nurs 2011;29:89. 21558032 10.1016/j.jvn.2011.03.002

[7] Bott-Kitslaar DM Saadiq RA McBane RD Efficacy and safety of rivaroxaban in patients with venous thromboembolism and active malignancy: a single-center registry. Am J Med 2016;129:615–9. 26797081 10.1016/j.amjmed.2015.12.025

[8] Nicklaus MD Ludwig SL Kettle JK Recurrence of malignancy-associated venous thromboembolism among patients treated with rivaroxaban compared to enoxaparin. J Oncol Pharm Pract 2018;24:185–9. 29284351 10.1177/1078155217690922

[9] Signorelli JR Gandhi AS Evaluation of rivaroxaban use in patients with gynecologic malignancies at an academic medical center: a pilot study. J Oncol Pharm Pract 2017;doi: 10.1177/1078155217739683. 10.1177/1078155217739683 29157146

[10] Liberati A Altman DG Tetzlaff J The PRISMA statement for reporting systematic reviews and meta-analyses of studies that evaluate health care interventions: explanation and elaboration. PLoS Med 2009;6:e1000100. 19621070 10.1371/journal.pmed.1000100 PMC2707010

[11] Alzghari SK Seago SE Garza JE Retrospective comparison of low molecular weight heparin vs. warfarin vs. oral Xa inhibitors for the prevention of recurrent venous thromboembolism in oncology patients: The Re-CLOT study. J Oncol Pharm Pract 2017;doi: 10.1177/1078155217718382. 10.1177/1078155217718382 28714376

[12] Prins MH Lensing AW Brighton TA Oral rivaroxaban versus enoxaparin with vitamin K antagonist for the treatment of symptomatic venous thromboembolism in patients with cancer (EINSTEIN-DVT and EINSTEIN-PE): a pooled subgroup analysis of two randomised controlled trials. Lancet Haematol 2014;1:e37–46. 27030066 10.1016/S2352-3026(14)70018-3

[13] Franchini M Bonfanti C Lippi G Cancer-associated thrombosis: investigating the role of new oral anticoagulants. Thromb Res 2015;135:777–81. 25743884 10.1016/j.thromres.2015.02.024

