# Supplementary figures and images for: Rivaroxaban versus enoxaparin for the prevention of recurrent venous thromboembolism in patients with cancer: A meta-analysis
Source: Medicine (Baltimore). 2018 Aug 3;97(31):e11384. doi: 10.1097/MD.0000000000011384 (PMC6081055; doi:10.1097/MD.0000000000011384)

**Supplementary Figure 1** Funnel plot of comparison: (A) VTE, (B) major bleeding and (C) mortality.


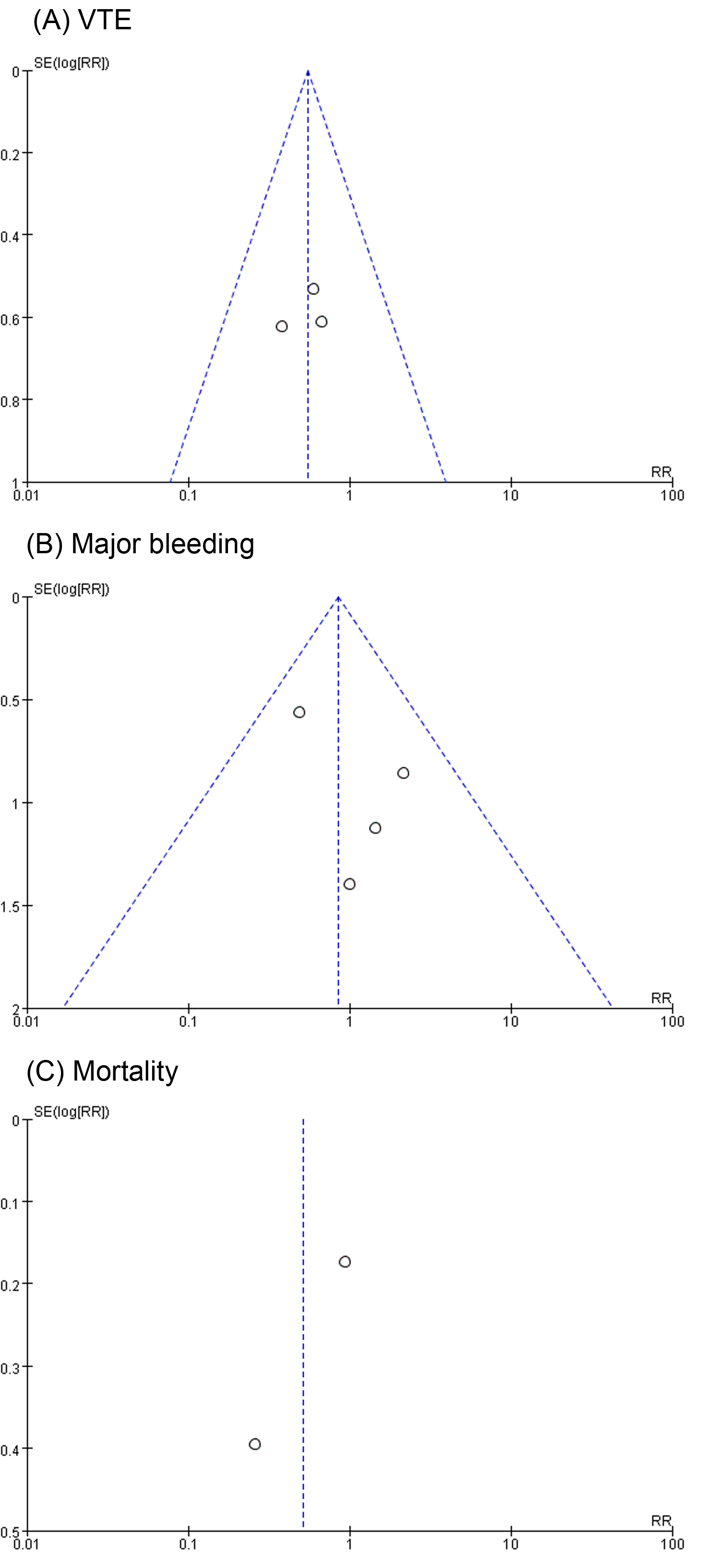

Supplement: Supplemental Digital Content [file medi-97-e11384-s001.doc]
